# Subclavian steal syndrome

**DOI:** 10.1002/ccr3.8561

**Published:** 2024-03-10

**Authors:** Durga Neupane, Sujan Kafle, Varsha Chhetri, Aakash Koirala

**Affiliations:** ^1^ B.P. Koirala Institute of Health Sciences Dharan Nepal

**Keywords:** angioplasty, dual antiplatelet therapy, stenting, subclavian steal

## Abstract

Early recognition and diagnosis of subclavian steal syndrome are vital to avoid unnecessary investigations and ensure appropriate management. This case highlights the significance of comprehensive evaluation, including bilateral blood pressure measurement, in patients with unilateral symptoms.

A 60‐year‐old woman with a history of hypertension presented to our hospital's Internal Medicine Outpatient Department with an 8‐month history of persistent left upper extremity numbness and dizziness, exacerbated by using her left hand for heavy lifting. She had never experienced chest pain, palpitations, tinnitus, hearing loss, or positional vertigo. Despite visiting multiple healthcare providers, including orthopedic and psychiatric specialists, and undergoing various diagnostic tests, her symptoms persisted. Notably, blood pressure measurements were not taken in both upper extremities during previous evaluations. A thorough physical examination revealed significant blood pressure differences between the two upper extremities, with the right upper extremity's BP being 170/100 mm of Hg and the left upper extremity's BP being 110/70 mm of Hg. In addition, the patient's left radial pulse was weak. Initial laboratory evaluations, such as a complete blood count, liver function test, renal function test, and fasting lipid profile, were within normal limits. Considering the absence of cardiac abnormalities on electrocardiogram and echocardiography, the suspicion of vascular occlusion emerged as a plausible cause for the patient's symptoms. Consequently, we referred her to a higher center for specialized evaluation. After referral, she was seen by a cardiologist, and he confirmed the blood pressure discrepancies between the two upper extremities. She underwent an angiography of the left subclavian artery, and it revealed a significant occlusion in the proximal left subclavian artery (Figure 1), thus confirming towards subclavian artery stenosis. Elective angioplasty of the left subclavian artery with stenting was scheduled for 1 week after the diagnosis. Following the angioplasty and stenting procedure, the angiogram showed complete restoration of blood flow in the left subclavian artery with visualization of anterograde blood flow in the left vertebral artery as well (Figure 2). The patient also experienced a notable improvement in her symptoms. The blood pressure discrepancy was corrected after the procedure. She was placed under dual antiplatelet therapy (Aspirin and Clopidogrel) and a statin after the procedure. She no longer complains of numbness in her left upper extremity or dizziness. She is on constant follow‐up with us.

**FIGURE 1:**
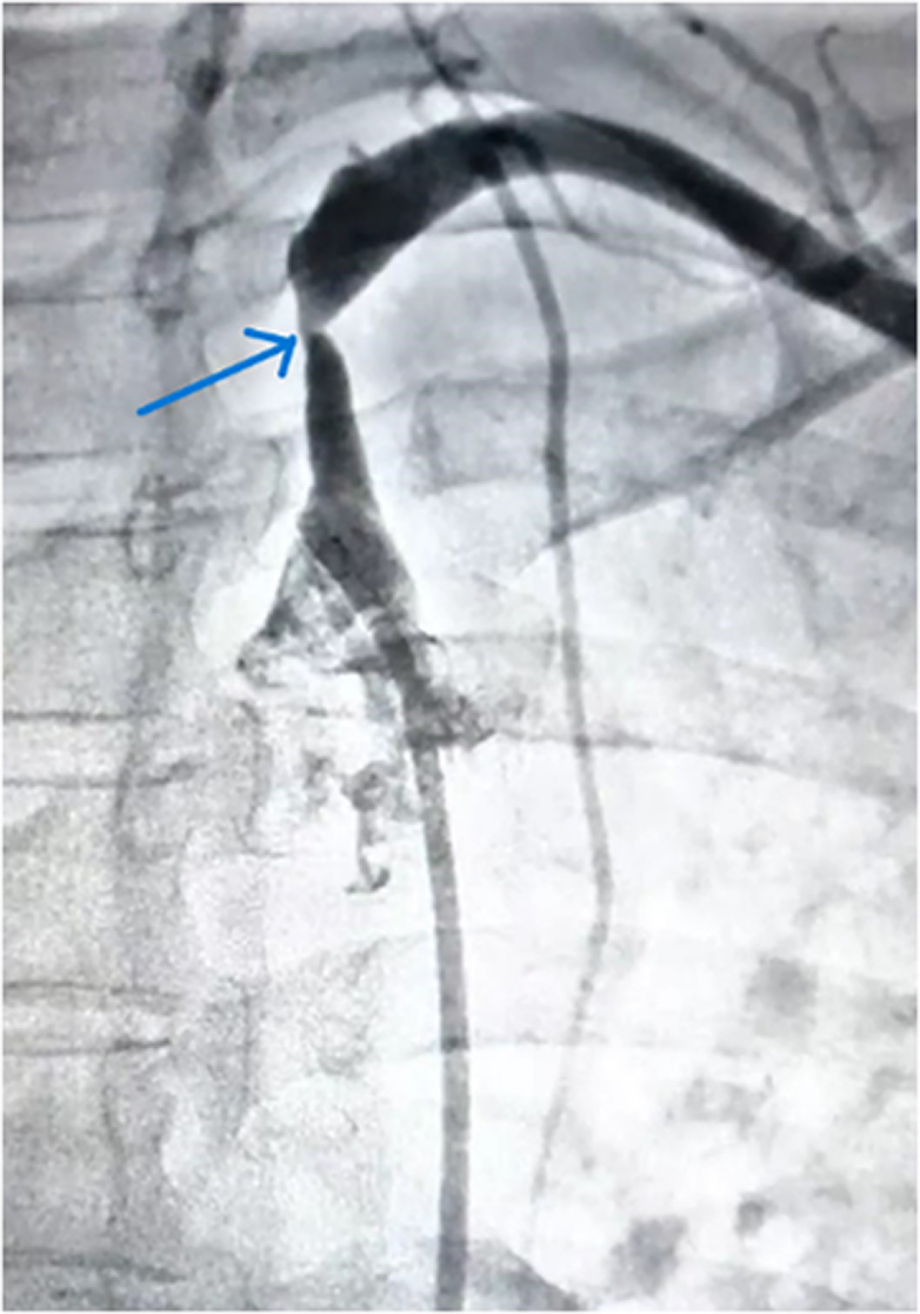
Angiogram shows tight stenosis in the left subclavian artery's proximal portion (blue arrow) and non‐visualization of the vertebral artery as a result of retrograde blood flow to the left upper limb even at rest.

**FIGURE 2:**
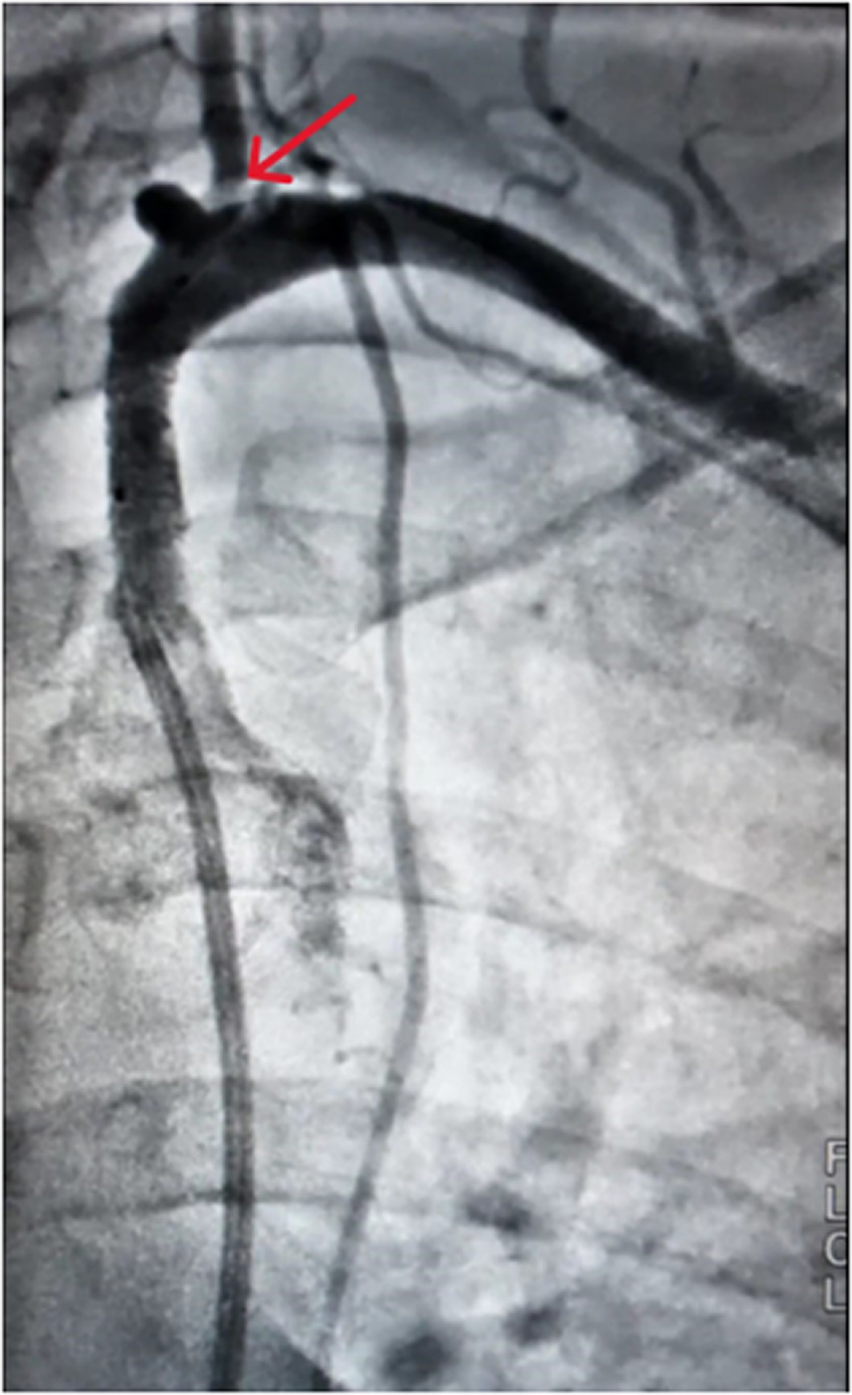
Angiogram shows complete opening of the stenosed subclavian arterial segment and establishment of anterograde flow in left vertebral artery (red arrow) which was not visualized on the pre‐angioplasty angiogram.

Subclavian steal syndrome (SSS) is a vascular condition characterized by the narrowing or blockage of prevertebral subclavian artery.^1^ Symptoms of SSS include exercise‐induced arm pain or fatigue (arm claudication), occasional coolness or paresthesias in the extremity. The diagnosis of SSS can be suggested by flow reversal in the ipsilateral vertebral artery on Doppler ultrasound. The subclavian stenosis or atresia can be **s**hown by catheter X‐ray angiography.^2^ Diagnosis can further be made by CT.

Angiography or MRI Angiography. Endovascular treatment, including balloon angioplasty and stenting, has become popular due to its minimally invasive nature and comparable outcomes.^3^ Open surgical bypass technique is another method of treatment.

Early recognition and diagnosis of SSS are vital to avoid unnecessary investigations and ensure appropriate management. This case highlights the significance of comprehensive evaluation, including bilateral blood pressure measurement, in patients with unilateral symptoms. Increased awareness among healthcare providers and timely referral to specialized centers can lead to successful revascularization, symptom resolution, and improved outcomes.

## AUTHOR CONTRIBUTIONS

**Durga Neupane:** Conceptualization; data curation; investigation; methodology; resources; supervision; validation; writing – original draft; writing – review and editing. **Sujan Kafle:** Conceptualization; data curation; investigation; methodology; resources; supervision; writing – original draft; writing – review and editing. **Varsha Chhetri:** Conceptualization; data curation; investigation; methodology; resources; writing – original draft; writing – review and editing. **Aakash Koirala:** Conceptualization; data curation; investigation; methodology; resources; supervision; validation; writing – original draft; writing – review and editing.

## FUNDING INFORMATION

None.

## CONFLICT OF INTEREST STATEMENT

None.

## ETHICS STATEMENT

Not required.

## CONSENT

Written informed consent was obtained from the patient for accompanying images.

## Data Availability

Relevant data is available in the manuscript.
